# Fall Prevention Interventions and Fracture Risk in Community-Dwelling Older Adults: A Systematic Review and Meta-Analysis

**DOI:** 10.3390/clinpract16030052

**Published:** 2026-02-28

**Authors:** Yazan Jumah Alalwani, Munira Abdullah Aldossari, Layan Adeeb Alzahrani, Nouf Ibrahim Alhatlani, Sarah Musaad Albarrak, Waleed Khalid Moosa, Raghad Ali Aloufi, Ibtisam Heji AlBader, Sadeem Khalid Almulhim, Nurah Jamel Alnbi, Leen Awad Alkahtani, Fatimah Mohammed Alsayoud, Ahmed Y. Azzam, Ghada Fouad Al Yousif

**Affiliations:** 1 College of Medicine, Imam Abdulrahman Bin Faisal University, Dammam 34212, Saudi Arabiawldashr@gmail.com (W.K.M.); ibtisamhalbader@gmail.com (I.H.A.); 2 College of Medicine, King Saud Bin Abdulaziz University for Health Sciences, Riyadh 11481, Saudi Arabia; 3 College of Medicine, Princess Nourah Bint Abdulrahman University, Riyadh 13412, Saudi Arabia; 4 College of Medicine, King Abdulaziz University, Jeddah 21589, Saudi Arabia; 5 College of Medicine, Hail University, Hail 55476, Saudi Arabia; saramusaid7654@gmail.com; 6 College of Medicine, Taibah University, Medina 42353, Saudi Arabia; 7 College of Medicine, King Faisal University, Hofuf 31982, Saudi Arabia; 8 College of Medicine, Vision College, Riyadh 11691, Saudi Arabia; nura.jamil99@gmail.com (N.J.A.);; 9 ASIDE Healthcare, Lewes, DE 19958, USA; 10 Family and Community Medicine, College of Medicine, Imam Abdulrahman Bin Faisal University, Dammam 34212, Saudi Arabia; gfalyousif@iau.edu.sa

**Keywords:** falls, fractures, elderly, geriatric, hip fractures

## Abstract

**Introduction:** Falls and subsequent fractures represent a major public health concern among older adults. While fall prevention interventions have demonstrated efficacy in reducing falls, their impact on fracture outcomes remains unclear. **Methods:** We conducted a systematic review and meta-analysis following PRISMA 2020 guidelines. We searched multiple databases up to 7 June 2025 for studies investigating fall prevention interventions and fracture outcomes in community-dwelling older adults. A primary outcome was hip fractures; secondary outcomes included any fractures, falls, and serious fall injuries. Risk ratios (RRs) with 95% confidence intervals (CIs) were calculated, using random-effects meta-analysis where appropriate. **Results:** Seventeen studies were included, spanning over 25,000 participants. Interventions included exercise programs, multifactorial approaches, medication optimization, and vitamin D supplementation. For hip fractures, only two randomized controlled trials (RCTs) reported extractable outcome data (12,489 participants; 132 events); both showed non-significant reductions favoring intervention (RR 0.80–0.87), precluding pooled meta-analysis. For any fractures, five studies (18,519 participants; 1343 events) demonstrated no significant effect (RR 0.91, 95% CI 0.72–1.14; *p*-value = 0.40) with significant heterogeneity (I^2^ = 65%). Fall prevention interventions significantly reduced falls across 14 studies. GRADE assessment indicated very low certainty for both hip fractures and any fractures due to limited studies, inconsistency, and imprecision. **Conclusions:** Current evidence suggests fall prevention interventions may reduce hip fractures but do not significantly prevent fractures overall. Despite consistent fall reduction, the translation to fracture prevention remains uncertain, highlighting the need for integrated interventions targeting both fall risk and bone health.

## 1. Introduction

Falls among older adults represent one of the most significant public health challenges of our aging society, affecting around one-third of community-dwelling adults aged 65 years and older annually. The consequences of falls extend far beyond immediate injury, including reduced quality of life, increased healthcare utilization, institutionalization, and significant economic burden on healthcare systems worldwide, with annual costs exceeding $50 billion in the United States alone. Among the most devastating outcomes of falls are fractures, especially hip fractures, which carry a one-year mortality rate of 20–30% and result in prolonged disability and loss of independence [1,2,3].

Understanding why falls do not always result in fractures is essential for evaluating prevention strategies. While falls are the leading cause of fractures in older adults—accounting for over 90% of hip fractures and a significant proportion of other osteoporotic fractures—not all falls result in fractures. Factors such as bone mineral density, fall characteristics, protective responses, and environmental conditions all impact whether a fall translates into a fracture. This complex, multifactorial relationship raises a critical question: do interventions that successfully prevent falls necessarily prevent fractures, or are additional bone-targeted strategies required [4,5]?

Previous studies have demonstrated the efficacy of various fall prevention interventions in reducing fall rates among community-dwelling older adults. Exercise programs with proven efficacy include balance training (static and dynamic exercises and Tai Chi), lower extremity strength training, and gait training, typically delivered 2–3 times weekly over 12–24 months through structured programs such as the Otago Exercise Programme. Multifactorial interventions combine individualized risk assessment with medication review (particularly psychotropic and cardiovascular agents), environmental modifications (home hazard removal, grab bar installation, and lighting improvements), and exercise components. Vitamin D supplementation targets fall prevention through its role in neuromuscular function, wherein deficiency impairs muscle strength and postural stability, with potential additional bone-protective effects via enhanced calcium absorption. While systematic reviews consistently demonstrate these interventions reduce falls, evidence for fracture prevention specifically remains less conclusive and scattered across studies with varying designs and populations [6,7,8,9,10,11].

Previous systematic reviews of fall prevention interventions have predominantly focused on fall outcomes, with fractures typically treated as secondary endpoints or analyzed in individual studies without pooled synthesis. This represents a critical evidence gap: while we have robust meta-analytic evidence that interventions reduce falls, we lack equivalent synthesis specifically addressing whether this fall reduction translates into clinically meaningful fracture prevention. The substantial heterogeneity in intervention types, study populations, and outcome definitions across studies necessitates a structured manner to synthesize the available evidence and assess the quality and certainty of findings related to fracture prevention [6,7,8,9,10,12,13].

Understanding whether fall prevention interventions effectively reduce fracture risk has important implications for practice, public health policy, and resource allocation. If these interventions could demonstrate significant fracture reduction benefits, it would strengthen the case for their widespread implementation and impact guidelines. Conversely, if the evidence suggests limited fracture prevention effects despite fall reduction, it may indicate the need for complementary strategies specifically targeting bone health and fracture prevention.

The objective of this systematic review and meta-analysis is to evaluate the effectiveness of fall prevention interventions in reducing fracture outcomes (specifically, the occurrence of fractures as reported in clinical trials) among community-dwelling older adults, with hip fractures as the primary outcome and any fractures, falls, and serious fall injuries as secondary outcomes. We note that this analysis focuses on observed fracture events within trials rather than changes in underlying fracture risk factors such as bone mineral density.

## 2. Materials and Methods

### 2.1. Study Design and Search Strategy

We conducted this systematic review and meta-analysis in accordance with the Preferred Reporting Items for Systematic Reviews and Meta-Analyses (PRISMA) 2020 guidelines [14]. This systematic review was not prospectively registered in PROSPERO, and no formal protocol document was prepared, which is acknowledged as a methodological limitation. However, eligibility criteria, outcomes, and analytical methods were determined by author consensus prior to the literature search and data extraction to minimize selective reporting bias. The study was conducted in accordance with the PRISMA 2020 guidelines (see PRISMA 2020 checklist in Supplementary Materials).

A dedicated literature search was conducted across multiple electronic databases from inception through 7 June 2025. The databases searched included MEDLINE (via PubMed), Scopus, Cochrane Central Register of Controlled Trials (CENTRAL), Google Scholar, and Web of Science. Google Scholar and Web of Science were included to maximize sensitivity and capture gray literature, conference proceedings, and studies indexed in journals not covered by traditional biomedical databases, consistent with recommendations for comprehensive systematic review searches.

The search strategy included the following key terms and concepts: (“fall prevention” OR “falls prevention” OR “fall reduction” OR “falls reduction”) AND (“exercise” OR “physical activity” OR “balance training” OR “strength training” OR “tai chi” OR “multifactorial intervention” OR “medication review” OR “medication optimization” OR “environmental modification” OR “home safety” OR “vitamin D” OR “calcium supplementation”) AND (“fracture” OR “hip fracture” OR “vertebral fracture” OR “wrist fracture” OR “osteoporotic fracture” OR “fragility fracture”) AND (“older adult” OR “elderly” OR “aged” OR “senior” OR “geriatric”) AND (“randomized controlled trial” OR “clinical trial” OR “RCT”). The search strategy was adapted appropriately for each database, utilizing database-specific controlled vocabulary terms and syntax. Additionally, we conducted manual searches of reference lists from included studies and relevant systematic reviews to identify any missed studies.

### 2.2. Eligibility Criteria

Studies were included if they met the following criteria: original studies investigating any fall prevention interventions in community-dwelling adults aged 65 years or older, with fracture outcomes reported as primary or secondary endpoints. Interventions of interest included exercise programs, multifactorial fall prevention strategies, medication optimization, environmental modifications, vitamin D supplementation, or combinations of these strategies. Studies were required to have a control group that received usual care, a placebo, or an alternative intervention, with follow-up periods of at least six months.

Exclusion criteria included studies focusing only on fall risk assessment without intervention; studies with insufficient data for meta-analysis; and non-original studies, such as reviews, systematic reviews, meta-analyses, case reports, or case series (if they included less than ten patients in their cohort), as well as editorials. Studies published in languages other than English were excluded due to resource limitations, which may have introduced selection bias.

### 2.3. Study Selection and Data Extraction

Two reviewers (Y.J.A. and M.A.A.) independently screened titles and abstracts of identified records using pre-defined eligibility criteria. Full-text articles of potentially relevant studies were then independently assessed for inclusion by both reviewers, with disagreements resolved through discussion and consensus or consultation with a third reviewer (A.Y.A).

Data extraction was performed by four reviewers (Y.J.A, L.A. Alzahrani, N.I.A, and S.M.A) working in pairs using a standardized data extraction form; this larger team was used to manage extraction volume while maintaining dual independent review. Extracted data included study characteristics, such as author information, publication year, country, and study design; participant demographics, including sample size, age, sex distribution, and baseline characteristics; intervention details, including type, duration, frequency, and delivery method; control group characteristics; outcome definitions and measurement methods; follow-up duration; and results for all relevant outcomes. Discrepancies were resolved through discussion and consensus. When data were unclear or missing, we attempted to contact study authors for clarification.

### 2.4. Quality Assessment and Risk of Bias

The methodological quality of included RCTs was assessed using the Cochrane Risk of Bias tool version 2 (RoB 2). We evaluated each study across five domains: randomization process, deviations from intended interventions, missing outcome data, measurement of outcomes, and selection of reported results. Each domain was rated as low risk, some concerns, or high risk of bias, with an overall risk of bias judgment assigned based on the individual domain assessments.

For non-randomized studies, when applicable, the Risk of Bias in Non-randomized Studies of Interventions (ROBINS-I) tool was utilized. Inter-rater agreement was calculated, and disagreements were resolved through discussion and consensus.

### 2.5. Statistical Analysis and Data Synthesis

Meta-analyses were performed using random-effects models with the DerSimonian–Laird method to account for anticipated heterogeneity between studies. Effect sizes were calculated as risk ratios (RRs) with 95% confidence intervals (CIs) for dichotomous outcomes. Statistical heterogeneity was assessed using the I^2^ statistic and Cochran’s Q test, with I^2^ values of 25%, 50%, and 75% representing low, moderate, and high heterogeneity, respectively.

Subgroup analyses were planned a priori based on intervention type, study design, participant characteristics, and risk of bias assessment. Sensitivity analyses were conducted to explore the robustness of findings by excluding studies with a high risk of bias or outlying results. The Hartung–Knapp–Sidik–Jonkman (HKSJ) adjustment was applied to random-effects models to provide more conservative 95% CIs, which is more appropriate when the number of studies is small. Leave-one-out analysis was performed by iteratively removing each study and recalculating the pooled estimate to assess the influence of individual studies on overall results. Publication bias was assessed using funnel plots for visual inspection; however, formal statistical testing using Egger’s test was only performed when ≥10 studies were available for a given outcome, as recommended by Cochrane guidance, since such tests have low power with fewer studies. Funnel plots were still constructed for outcomes with fewer than ten studies to allow visual assessment, with the caveat that interpretation is limited. When fewer than three studies were available for a given outcome, qualitative synthesis was performed rather than pooled meta-analysis, as meaningful assessment of heterogeneity and robustness requires a minimum of three studies.

Clinical significance was evaluated by calculating the number needed to treat, absolute risk reduction, and relative risk reduction where appropriate. All statistical analyses were performed using RStudio version 2025.09 with R version 4.4.2, utilizing the meta package (version 8.2-1) and metafor package (version 4.8-0) for meta-analytical computations. Statistical significance was set at a *p*-value less than 0.05.

### 2.6. Certainty of Evidence Assessment

The certainty of evidence for each outcome was assessed using the Grading of Recommendations Assessment, Development and Evaluation (GRADE) approach. Evidence was rated as high, moderate, low, or very low certainty based on the consideration of risk of bias, inconsistency, indirectness, imprecision, and publication bias. The GRADE assessment informed the strength of conclusions and recommendations derived from the review findings.

## 3. Results

### 3.1. Study Selection and Characteristics

**Figure 1:**
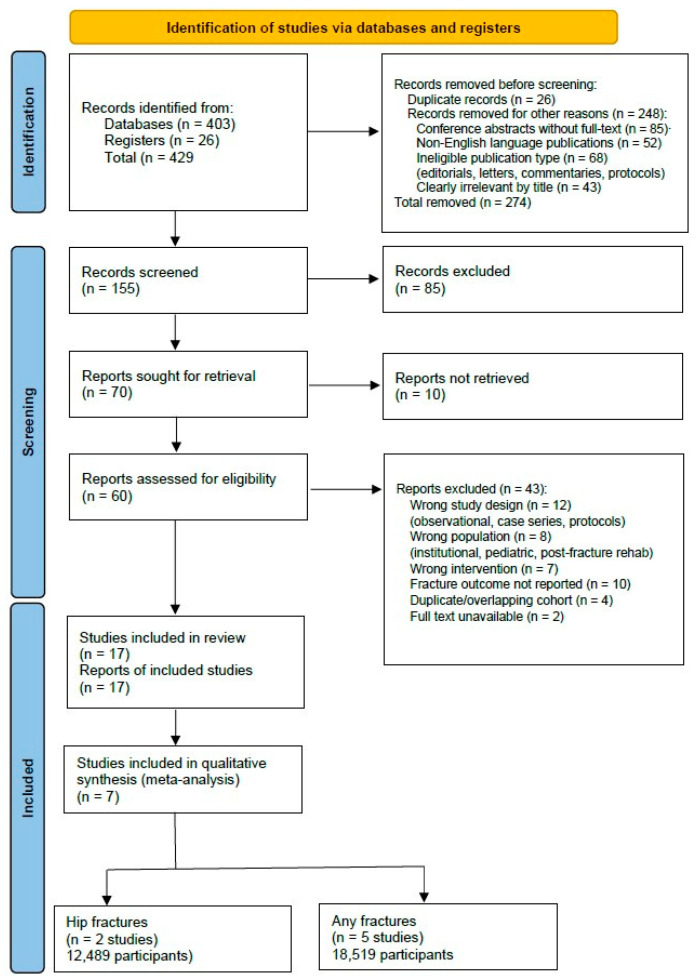
PRISMA flowchart diagram.

The literature search identified 429 records (403 from database searches and 26 from registers). After removing 26 duplicate records, 248 records were removed before screening for the following reasons: conference abstracts without retrievable full-text (*n* = 85); non-English language publications (*n* = 52); ineligible publication types, including editorials, commentaries, and letters (*n* = 68); and clearly irrelevant titles (*n* = 43). The remaining 155 records were screened by title and abstract. Following screening, 85 records were excluded for not meeting the inclusion criteria, leaving 70 reports for full-text retrieval. Of these, 10 reports could not be retrieved, leaving 60 reports for full-text assessment. After full-text assessment, 43 reports were excluded for the following reasons: no fracture outcomes reported (*n* = 18), wrong population or setting (*n* = 12), wrong intervention type (*n* = 8), and insufficient data for meta-analysis (*n* = 5). This resulted in 17 studies meeting the inclusion criteria for this systematic review and meta-analysis (Figure 1).

Table 1 presents the characteristics and participant demographics of the 17 included studies, spanning publication years from 1997 to 2024. The studies were conducted across multiple countries, including the United States (three studies), the United Kingdom (three studies), New Zealand (five studies), Germany (two studies), and single studies, each from Italy, Thailand, Canada, and the Netherlands. Study designs included individual RCTs (12 studies), cluster RCTs (four studies), and one follow-up study ([15], 2-year extension), with sample sizes ranging from 93 participants in [15] to 9803 participants in [16].

The mean age of participants across studies ranged from 69.0 years in Boongird et al., 2017 [21] to 84.1 years in Campbell et al., 1999 [15] (2-year follow-up), with several studies specifying inclusion criteria of adults aged 65 or 70 years or older. Female participation varied considerably, ranging from 24% in [26] (which recruited predominantly from a male British doctor’s register) to 100% in studies by Campbell et al. that specifically enrolled women only. Studies were conducted in both primary care and community settings, with follow-up durations varying from six months to five years. Primary endpoints included falls, fractures, medically treated falls, and serious fall injuries, reflecting the diverse focus areas within fall prevention evidence.

### 3.2. Intervention Characteristics and Components

Table 2 details the intervention characteristics and components across the included studies. The interventions were categorized into several main types: exercise interventions (eight studies), multifactorial interventions (five studies), medication optimization (one study), vitamin D supplementation (one study), and combined exercise plus medication or multifactorial approaches (two studies). Exercise interventions included targeted fall prevention programs, home-based exercise programs, and complex exercise interventions delivered through primary care settings.

Multifactorial interventions usually included multiple components, such as exercise, medication review, environmental assessment, and education. The medication optimization study by Phelan et al., 2024 [17] specifically focused on CNS-active medication reduction through patient education and clinician decision support. Delivery methods varied considerably, including home-based programs, primary care settings, community-based interventions, and postal mail combined with electronic health record messaging. Follow-up durations ranged from six months to five years, although many studies did not specify intervention duration clearly. Adherence rates were mostly underreported across most studies, limiting assessment of intervention fidelity.

### 3.3. Primary and Secondary Outcomes Measurement

Table 3 summarizes the primary and secondary outcome measurements across the included studies. Primary outcomes varied, with falls being the most common primary endpoint (eight studies), followed by fractures as a primary endpoint (two studies: Bruce et al., 2021 [16] and Trivedi et al., 2003 [26]) and serious fall injuries (one study). Fall definitions, when specified, generally included unintentional events resulting in the person coming to rest on the ground or lower level, though several studies did not provide explicit fall definitions.

Fracture ascertainment methods included medical record reviews, electronic health records, and administrative databases, with some studies focusing on fragility fractures or fractures resulting from falls. Hip fractures were consistently defined using ICD-10 coding systems where specified. Data sources included health plan electronic records, primary care electronic records, medical records, and hospital records. The heterogeneity in outcome definitions and measurement methods across studies represents a major methodological limitation that threatens the validity of pooled estimates and limits the interpretability of findings. This heterogeneity reflects fundamental inconsistencies in how falls and fractures are defined, ascertained, and reported across the fall prevention literature.

### 3.4. Quality Assessment and Risk of Bias

Table 4 presents the quality assessment and risk of bias evaluation for all included studies using the Cochrane Risk of Bias tool version 2. The majority of studies demonstrated adequate randomization and allocation concealment, with 13 studies rated as low risk for random sequence generation and ten studies achieving low risk for allocation concealment. However, blinding of participants presented a consistent challenge across intervention studies, with 15 studies rated as high risk due to the inherent difficulty of blinding behavioral and exercise interventions; Trivedi et al., 2003 [26] achieved low risk through placebo-controlled vitamin D supplementation.

Blinding of outcome assessors was more variable, with seven studies achieving low risk, three studies showing some concerns, and seven studies having unclear risk of bias. Incomplete outcome data were generally well-handled, with nine studies achieving low risk ratings. Selective reporting was adequately addressed in most studies, with ten studies rated as low risk. Overall risk of bias assessment revealed one study with low risk, ten studies with some concerns, and six studies with high risk of bias. The high risk ratings were primarily driven by the lack of participant blinding and unclear reporting of methodological details in several studies.

### 3.5. Meta-Analysis of Hip Fractures

Three studies initially appeared to report hip fracture outcomes; however, Robertson et al. (2001a) [28] was not included in this analysis as the study reported serious injuries as a composite endpoint without disaggregation by fracture site. Given the limited number of eligible studies, qualitative synthesis was performed rather than pooled meta-analysis.

Two RCTs provided extractable hip fracture data (12,489 participants; 132 events). Given this limited number, qualitative synthesis was performed rather than pooled meta-analysis.

Trivedi et al. (2003) [26] evaluated four-monthly vitamin D3 supplementation in 2686 community-dwelling adults over five years. Hip fractures occurred in 21 versus 24 participants (RR 0.87, 95% CI 0.49–1.56). Bruce et al. (2021) [16] compared exercise and multifactorial interventions with advice alone in 9803 participants over 18 months. Hip fractures occurred in 54 versus 33 participants (RR 0.80, 95% CI 0.52–1.23).

Both studies showed non-significant reductions favoring intervention (RR 0.80–0.87) with wide confidence intervals crossing the null. The differing intervention types and vitamin D versus exercise-based approaches limited direct comparison. Sensitivity analyses and publication bias assessment were not performed due to insufficient studies.

### 3.6. Meta-Analysis of Any Fractures

Five studies reported any fractures with group-specific data (18,519 participants; 1343 events). Robertson et al. (2001a) [28] was excluded as fracture counts were reported only as a combined total across treatment groups without disaggregation. Marrocco et al. (2023) [18] was excluded as a statistical outlier (RR 2.32), showing a harm direction inconsistent with other studies.

**Figure 2:**
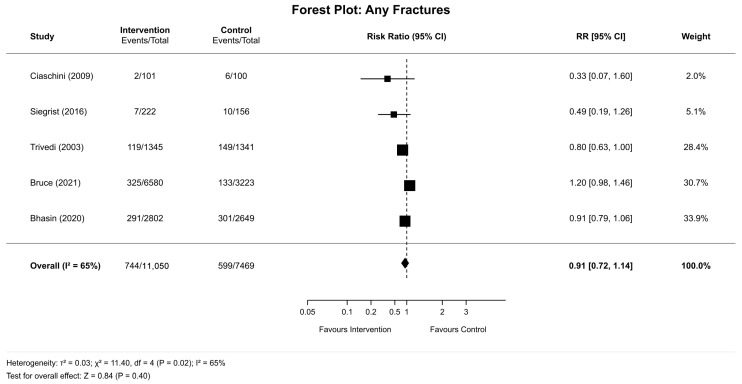
Forest plot of any fractures [16,20,22,24,26].

The pooled estimate showed no significant effect (RR 0.91, 95% CI 0.72–1.14; *p*-value= 0.40) with significant heterogeneity (I^2^ = 65%; τ^2^ = 0.03; Q = 11.40, df = 4, *p*-value= 0.02) (Figure 2). Individual study results: Ciaschini et al., 2009 [24] (RR 0.33, 95% CI 0.07–1.60; weight 2.0%), Siegrist et al., 2016 [22] (RR 0.49, 95% CI 0.19–1.26; weight 5.1%), Trivedi et al., 2003 [26] (RR 0.80, 95% CI 0.63–1.00; weight 28.4%), Bruce et al., 2021 [16] (RR 1.20, 95% CI 0.98–1.46; weight 30.7%), and Bhasin et al., 2020 [20] (RR 0.91, 95% CI 0.79–1.06; weight 33.9%). Bruce et al., 2021 [16] was the only study showing a harm direction. Absolute risk reduction was 0.75% (95% CI −1.16% to 2.33%) with a number needed to treat of 134 (95% CIs crosses null).

### 3.7. GRADE Evidence Assessment

Table 5 presents the GRADE evidence assessment. For hip fractures (12,489 participants; 132 events; 2 RCTs), certainty was rated very low due to very serious imprecision and inability to assess consistency with only two studies. Both studies showed non-significant reductions favoring intervention (RR 0.80–0.87).

For any fractures (18,519 participants; 1343 events; 5 studies), certainty was very low due to serious risk of bias, very serious inconsistency (I^2^ = 65%), and serious imprecision (RR 0.91, 95% CI 0.72–1.14). For falls (25,000+ participants; 14 studies), certainty was low due to serious risk of bias, inconsistency, and possible publication bias, though evidence suggests interventions may reduce falls. Serious fall injuries (8000+ participants; 3 studies) received low certainty due to serious inconsistency, indirectness, and imprecision.

### 3.8. Subgroup and Sensitivity Analysis

Sensitivity analyses for hip fractures were not performed as only two studies provided extractable data, precluding meaningful assessment of robustness.

**Figure 3:**
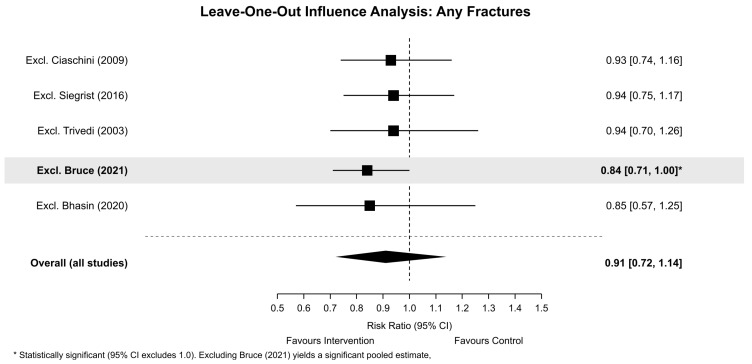
Any fractures leave-one-out influence plot. The grey square represents the overall effect estimate, the dotted line indicates the line of no effect, and bold format indicates statistically significant results [16,20,22,24,26].

For any fractures, HKSJ adjustment resulted in wider 95% CIs (RR 0.91, 95% CI 0.63–1.31), appropriately reflecting uncertainty. Leave-one-out analysis (Figure 3) revealed that excluding Bruce et al., 2021 [16] resulted in a statistically significant pooled estimate (RR 0.84, 95% CI 0.71–1.00; I^2^ = 18.7%), indicating sensitivity to this single influential study showing a harm direction. Excluding other studies did not materially alter conclusions: Ciaschini et al., 2009 [24] (RR 0.93, 95% CI 0.74–1.16), Siegrist et al., 2016 [22] (RR 0.94, 95% CI 0.75–1.17), Trivedi et al., 2003 [26] (RR 0.94, 95% CI 0.70–1.26), and Bhasin et al., 2020 [20] (RR 0.85, 95% CI 0.57–1.25). Including Marrocco et al., 2023 [18] moved the estimate toward null (RR 0.97, 95% CI 0.76–1.23; I^2^ = 65%), supporting its exclusion as a statistical outlier.

### 3.9. Publication Bias Assessment

Publication bias assessment for hip fractures was not performed as only two studies provided extractable data, which is insufficient for funnel plot interpretation.

**Figure 4:**
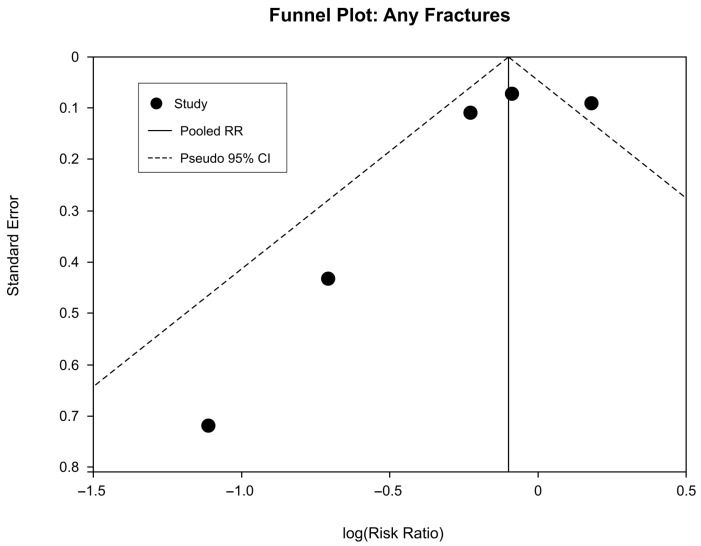
Any fractures funnel plot for publication bias assessment.

For any fractures, the funnel plot included five studies with a pooled RR of 0.91 (log RR = −0.10) (Figure 4). Visual inspection revealed studies distributed across both sides of the pooled estimate, with smaller studies showing greater dispersion as expected. Formal statistical testing was not performed, given the limited number of studies (*n* < 10), as such tests have low power and may yield misleading results. The observed heterogeneity (I^2^ = 65%) may also contribute to apparent asymmetry independent of publication bias. No obvious visual evidence of major publication bias was detected; however, definitive assessment remains limited.

## 4. Discussion

Falls among community-dwelling older adults represent a public health challenge with far-reaching consequences extending beyond immediate physical injury. The cascade of events following a fall often includes fractures, especially hip fractures, which are associated with increased mortality, prolonged disability, loss of independence, and added burden from healthcare costs. Understanding this relationship is important, given the aging global population and the increasing burden of fall-related injuries on healthcare systems around the world [32,33,34,35].

While previous studies from the literature have demonstrated the efficacy of various fall prevention interventions in reducing fall rates among older adults, the translation of these benefits to fracture prevention has remained less clear. This disconnect raises important questions and considerations about whether interventions designed mainly to prevent falls necessarily provide adequate protection against fractures, which depend on multiple factors, including bone mineral density, fall characteristics, protective responses, and environmental conditions. Our systematic review and meta-analysis aimed to address this gap by focusing on and investigating the effectiveness of fall prevention interventions in reducing fracture risk among community-dwelling older adults [36,37,38,39].

Based on our analyses, we found several important implications. For hip fractures as the primary outcome, only two RCTs provided extractable data, precluding pooled meta-analysis. Both studies showed non-significant reductions favoring intervention (RR 0.80–0.87), but wide confidence intervals and the inability to synthesize findings quantitatively resulted in very low certainty evidence. Given the limited data, clinical significance measures such as absolute risk reduction and number needed to treat could not be reliably calculated.

The analysis of any fractures demonstrated very low certainty evidence showing no significant effect of fall prevention interventions (RR 0.91, 95% CI 0.72–1.14), with significant heterogeneity (I^2^ = 65%) indicating considerable variation in treatment effects across studies. Individual study results varied, with some studies showing protective effects while others demonstrated increased fracture risk, highlighting the complexity and inconsistency in fracture prevention outcomes.

In stark contrast to the fracture outcomes, fall prevention interventions demonstrated effectiveness in reducing falls across 14 studies, despite the evidence being rated as low certainty due to risk of bias and inconsistency. This finding highlights a significant and concerning disconnect; while these interventions successfully reduce fall occurrence, this reduction does not reliably translate into fracture prevention. For serious fall injuries, our analysis of three studies including over 8000 participants showed low certainty evidence for a possible promising benefit; however, this was limited by heterogeneous outcome definitions and measurement estimations.

There are multiple implications and considerations from our findings that warrant keeping an eye on, add to the current evidence in the literature, and should be considered for future studies. The observation that effective fall reduction does not translate to fracture prevention suggests that fall prevention and fracture prevention may require peculiar, however, possibly complementary, approaches. This finding aligns with emerging recognition in geriatric medicine that bone health and fall prevention represent related but separate therapeutic targets [37,40,41,42,43,44].

Our results are in agreement with previous studies that have found and demonstrated variable effects of fall prevention interventions on fracture outcomes [8,15,43,45,46,47,48,49]. However, our study provides a further step in advancing the evidence by focusing on fracture endpoints and the utilization of a GRADE methodology to assess evidence certainty. The very low certainty evidence for hip fracture reduction, based on only two RCTs with non-significant individual results, suggests a possible benefit that requires confirmation in larger studies, especially given the devastating consequences of hip fractures in older adults.

The significant heterogeneity observed in the any fractures analysis reflects the different nature of both interventions and study populations included in fall prevention studies. This heterogeneity may also indicate that certain types of fall prevention interventions or specific population subgroups may have greater fracture prevention benefits than others. The variation in individual study results, ranging from protective effects to increased fracture risk, highlights the need for more targeted and personalized approaches to fracture prevention.

Our findings suggest that while exercise programs, multifactorial interventions, medication optimization, and vitamin D supplementation effectively reduce falls, they may not adequately address the bone health components necessary for fracture prevention. Exploratory examination of our data by intervention type revealed that vitamin D supplementation ([26]: RR 0.87 for hip fractures, RR 0.80 for any fractures) showed numerically favorable effects, while exercise-based interventions showed more variable results ([15]: RR 0.80 for hip fractures but RR 1.20 for any fractures). These patterns suggest that interventions incorporating bone-protective components such as vitamin D supplementation or high-intensity resistance training may offer greater fracture prevention potential than fall prevention alone, though formal subgroup analyses were precluded by the small number of studies. This observation has important implications for clinical practice, suggesting that structured and focused approaches targeting both fall risk and bone health may be necessary to achieve meaningful fracture prevention outcomes.

Several important limitations must be acknowledged in interpreting our findings. First, the small number of studies reporting fracture outcomes, especially for hip fractures, which included only two RCTs with extractable data, limited the validity of our conclusions and precluded pooled meta-analysis for this outcome. This limitation reflects the historical focus on fall outcomes rather than fracture endpoints in fall prevention studies, resulting in fractures often being treated as secondary outcomes with insufficient power to detect differences.

Second, significant heterogeneity in intervention types, delivery methods, duration, and intensity across studies made it challenging to identify which specific components or approaches might be most effective for fracture prevention. The interventions ranged from simple vitamin D supplementation to complex multifactorial programs, each with different mechanisms for fracture prevention. In addition to that, adherence rates were poorly reported across most studies, limiting our ability to assess intervention fidelity and dose–response relationships.

Third, the quality assessment revealed significant methodological concerns, with most studies rated as having “some concerns” or “high risk” of bias, which is mainly due to the inherent difficulty of blinding participants to behavioral interventions and unclear reporting of methodological details. The inability to blind participants to exercise and behavioral interventions represents a challenge in fall prevention studies that may introduce performance bias.

Fourth, outcome measurement and ascertainment methods varied in the included studies, with different definitions for falls, fractures, and serious injuries. Some studies relied on self-reported outcomes, while others used medical records or administrative databases, which could have introduced measurement bias and limited the comparability of results. The heterogeneous follow-up periods, ranging from six months to five years, further complicated the synthesis of findings.

Also, publication bias remains a possible underlying concern; despite this, we attempted to perform statistical testing for publication bias assessment, but it was limited by the small number of studies. The majority of studies from high-income countries with well-developed healthcare systems may also limit the generalizability of findings to other healthcare settings and populations with different baseline fracture risks.

Based on our findings and identified limitations, several important recommendations are warranted for future studies and upcoming investigations. First, there is a need for larger, adequately powered clinical trials specifically designed with fracture prevention as the primary endpoint. These studies should include sufficient follow-up periods to capture meaningful fracture outcomes and should be powered to detect clinically important differences in fracture rates.

Future studies shall also focus on developing and testing integrated interventions that simultaneously target fall risk and bone health. This might include combining the current standard fall prevention strategies based on current evidence from the literature, with bone-specific interventions such as resistance training, calcium and vitamin D supplementation, and properly selected pharmacological bone health treatments. Such integrated approaches should be tested using factorial trial designs to understand the individual and synergistic effects of different intervention components.

The standardization of outcome definitions and measurement methods is also of significant importance. Developing consensus definitions for fall-related fractures, serious fall injuries, and other key outcomes will help and assist in developing better evidence for more meaningful meta-analyses. In addition, the consistent use of validated assessment tools and standardized follow-up periods would improve the quality and comparability of future studies.

From a clinical practice perspective, our findings suggest that healthcare providers should not assume that effective fall prevention automatically confers adequate fracture protection. Instead, a structured and detailed geriatric assessment should include the separate evaluation and management of both fall risk and bone health. This might include implementing dual-focus interventions that address both domains simultaneously, especially for high-risk older adults.

Future trials and studies should explore better-focused and tailored approaches to identify which older adults are most likely to benefit from specific types of fall and fracture prevention interventions. This might include developing risk stratification tools that consider both fall risk factors and bone health parameters to guide personalized intervention selection. Also, investigating the timing, intensity, and duration of interventions across different risk groups will help maximize the safety and effectiveness of fall and fracture prevention programs.

## 5. Conclusions

Our systematic review reveals a critical gap in geriatric care; while fall prevention interventions effectively reduce falls, they do not reliably prevent fractures. This disconnect challenges the assumption that reducing falls automatically protects against fractures, an assumption that has shaped clinical guidelines and resource allocation for decades.

For hip fractures, only two RCTs provided extractable data, precluding pooled meta-analysis; both showed non-significant reductions favoring intervention (RR 0.80–0.87; very low certainty). For any fractures, pooled analysis of five studies showed no significant effect (RR 0.91, 95% CI 0.72–1.14; very low certainty) with significant heterogeneity (I^2^ = 65%). Given the limited evidence base and 95% CIs crossing null, current fall prevention strategies alone cannot be expected to meaningfully reduce fracture burden at the population level.

These findings carry important implications for healthcare policy and clinical practice. Fall prevention and fracture prevention should be conceptualized as complementary but distinct focused goals. Clinical guidelines should recommend dual assessment of both fall risk and bone health in older adults, with targeted interventions for each. Future trials must be specifically powered for fracture endpoints rather than treating fractures as secondary outcomes and should report fracture data disaggregated by treatment group and anatomical site to enable calculable statistical synthesis.

Ultimately, this study calls for a change from fall-centric to fracture-centric prevention strategies that integrate bone health optimization alongside fall risk reduction. Healthcare providers should not assume that successful fall prevention automatically confers adequate fracture protection. Only through integrated approaches simultaneously targeting both fall risk and bone health can we expect to reduce the devastating burden of fractures in our aging population.

## Figures and Tables

**Table 1:** Study characteristics and participant demographics.

Study Citation	Country	Study Design	Sample Size (I/C)	Mean Age (SD)	Female %	Setting	Follow-Up Duration	Primary Endpoint
Phelan et al., 2024 [17]	USA	Cluster RCT	1106/1261	70.6 (7.6)	63%	Primary care	24.8 months	Medically treated falls
Marrocco et al., 2023 [18]	Italy	RCT	875/882	77.5 (7.2)	63.5%	Primary care	Not specified	Falls and injuries
Hentschke et al., 2021 [19]	Germany	Cluster RCT	222/156	78.1 (5.6)	68%	Primary care	24 months	Falls
Bruce et al., 2021 [16]	UK	Cluster RCT	6580/3223	77.2 (5.4)	66.3%	Primary care	18 months	Falls and fractures
Bhasin et al., 2020 [20]	USA	RCT	2802/2649	79.9 (5.2)	62%	Community	Not specified	Serious fall injuries
Boongird et al., 2017 [21]	Thailand	RCT	218/219	69.0 (3.0)	77.8%	Primary care	12 months	Falls
Siegrist et al., 2016 [22]	Germany	Cluster RCT	222/156	77.9 (5.4)	68%	Primary care	Not specified	Falls
Gawler et al., 2016 [23]	UK	RCT	457 + 387/412 ‡	73.0 (5.2)	62%	Primary care	Not specified	Falls
Ciaschini et al., 2009 [24]	Canada	RCT	101/100	78.6 (6.2)	72%	Community	6 months	Falls risk management
Shumway-Cook et al., 2007 [25]	USA	RCT	226/227	75.0 (5.8)	73%	Community	Not specified	Falls and risk factors
Trivedi et al., 2003 [26]	UK	RCT	1345/1341	74.8 (4.4)	24%	Community	5 years	Fractures and mortality
Robertson et al., 2001 (Trial II) [27]	New Zealand	RCT	330/120	80.9 (3.9)	68%	Community	Not specified	Falls
Robertson et al., 2001 (Trial I) [28]	New Zealand	RCT	121/119	80.4 (3.7)	70%	Community	Not specified	Falls
van Haastregt et al., 2000 [29]	Netherlands	RCT	159/157	77.2 (5.2)	68%	Community	Not specified	Falls and mobility
Campbell et al., 1999 (Psychotropic) [30]	New Zealand	RCT	48/45 †	74.5 (3.6)	100%	Community	Not specified	Falls
Campbell et al., 1999 (2-year) [15]	New Zealand	Follow-up study	71/81	84.1 (3.1)	100%	Community	2 years	Falls
Campbell et al., 1997 [31]	New Zealand	RCT	116/117	79.5 (3.9)	100%	Community	Not specified	Falls

**Abbreviations:** RCT = Randomized Controlled Trial; I = Intervention group; C = Control group; SD = Standard Deviation. **Notes: ‡** Gawler 2016 was a 3-arm trial: Otago Exercise Programme (OEP) *n* = 457, Falls Management Exercise (FaME) *n* = 387, Control *n* = 412 (total *n* = 1256); † Campbell 1999 Psychotropic used a 2 × 2 factorial design (medication withdrawal vs. continued medication × exercise vs. no exercise); *n* = 93 total; sample sizes represent medication withdrawal (48) vs. continued medication (45) groups.

**Table 2:** Intervention characteristics and components.

Study Citation	Intervention Category	Specific Components	Duration/ Frequency	Delivery Method	Control Group
Phelan et al., 2024 [17]	Medication optimization	CNS-active medication reduction via patient education + clinician decision support	Delivered at baseline (single intervention, 12-month follow-up)	Postal mail + EHR messaging	Usual care
Marrocco et al., 2023 [18]	Multifactorial intervention	Multicomponent primary care intervention for fall prevention	12 months; monthly contact monitoring; 1–2 daily exercise sessions recommended	Primary care setting	Usual care
Hentschke et al., 2021 [19]	Exercise intervention	Targeted fall prevention program in primary care setting	24 months intervention period	Primary care setting	Not specified
Bruce et al., 2021 [16]	Exercise + Multifactorial	Exercise program + Multifactorial falls prevention program (MFFP)	6-month intervention; 18-month follow-up; individual/group sessions with therapist	Primary care	Advice leaflet only
Bhasin et al., 2020 [20]	Multifactorial intervention	Multifactorial strategy to prevent serious fall injuries	Ongoing individualized intervention; median 2.7 years follow-up (up to 3.5 years)	Community setting	Enhanced usual care
Boongird et al., 2017 [21]	Exercise intervention	Simple home-based exercise program	12 months; home-based exercises	Home-based	Control group
Siegrist et al., 2016 [22]	Exercise intervention	Targeted complex exercise intervention	16-week group exercise + 12-week home exercise; 12-month follow-up	Primary care setting	Control group
Gawler et al., 2016 [23]	Exercise intervention	ProAct65+ exercise intervention trial	24 weeks; FaME: weekly classes + home exercises; OEP: 3×/week home exercises	Primary care setting	Control group
Ciaschini et al., 2009 [24]	Multifactorial intervention	Community-based intervention to optimize falls risk management	6-month intervention; 12-month follow-up	Community-based	Usual care
Shumway-Cook et al., 2007 [25]	Multifactorial intervention	Community-based multifactorial intervention on falls and fall risk factors	12 months; 3×/week group exercise; 6 h fall prevention education	Community-based	Control group
Trivedi et al., 2003 [26]	Vitamin D supplementation	Four-monthly oral vitamin D3 (cholecalciferol) supplementation	5 years; four-monthly oral vitamin D3 dosing	Oral supplementation	Placebo
Robertson et al., 2001 (Trial II) [27]	Exercise intervention	Nurse-delivered home exercise program	12 months; 3×/week, 30 min home exercises; 5 home visits by nurse	Home-based, nurse-delivered	Control group
Robertson et al., 2001 (Trial I) [28]	Exercise intervention	Nurse-delivered home exercise program	12 months; 3×/week, 30 min home exercises; 5 home visits by nurse	Home-based, nurse-delivered	Control group
van Haastregt et al., 2000 [29]	Multifactorial intervention	Program of multifactorial home visits on falls and mobility	18 months; 5 home visits over 1 year by community nurse	Home visits	Control group
Campbell et al., 1999 (Psychotropic) [30]	Exercise + Medication	Psychotropic medication withdrawal + home-based exercise program	12 months; 3×/week home exercises + psychotropic medication withdrawal	Home-based	Control group
Campbell et al., 1999 (2-year) [15]	Exercise intervention	Home-based exercise program (2-year follow-up study)	2 years; 3×/week, 30 min home exercises (Otago Exercise Programme)	Home-based	Control group
Campbell et al., 1997 [31]	Exercise intervention	General practice program of home-based exercise to prevent falls	12 months; 3×/week, 30 min progressive home exercises; 4 home visits by nurse	Home-based, primary care	Control group

**Table 3:** Primary and secondary outcomes measurement.

Study Citation	Primary Outcome	Secondary Outcomes	Fall Definition	Fracture Ascertainment	Data Source
Phelan et al., 2024 [17]	Medically treated falls	Medication discontinuation, sustained discontinuation, dose reduction	Falls for which medical attention sought (ICD-10 codes S, T, M, W)	Not applicable	Health plan electronic records
Marrocco et al., 2023 [18]	Falls and fall-related injuries	Fracture consequent to fall	Not specified	Fractures resulting from falls	Not specified
Hentschke et al., 2021 [19]	Falls	Not specified	Not specified	Not applicable	Not specified
Bruce et al., 2021 [16]	Falls and fractures	Time to first fracture, wrist/forearm fractures	Not specified	Proximal femoral (hip) fractures defined by ICD-10 code S72	Primary care electronic records
Bhasin et al., 2020 [20]	Serious fall injuries	Fractures (excluding thoracic/lumbar vertebrae)	Serious fall injury requiring medical attention	Adjudicated serious fall injuries, fractures specified in supplement	Medical records + supplement
Boongird et al., 2017 [21]	Falls	Not specified	Not specified	Not applicable	Not specified
Siegrist et al., 2016 [22]	Falls	Not specified	Not specified	Not applicable	Not specified
Gawler et al., 2016 [23]	Falls	Not specified	Not specified	Not applicable	Not specified
Ciaschini et al., 2009 [24]	Fall risk management	Documented fragility fracture	Not specified	Non-pathological fracture of vertebrae, hip or wrist	Medical records
Shumway-Cook et al., 2007 [25]	Fall and fall risk factors	Not specified	Not specified	Not applicable	Not specified
Trivedi et al., 2003 [26]	Fractures and mortality	Hip fractures, any fractures	Not applicable	Hip fractures defined as “Hip”, any fractures documented	Medical records
Robertson et al., 2001 (Trial II) [27]	Falls	Fractures (8 total falls resulted in fractures)	Not specified	Falls resulting in fractures, no site breakdown	Not specified
Robertson et al., 2001 (Trial I) [28]	Falls	Serious injury, including fractures	Not specified	Serious injury included falls resulting in fracture	Hospital and GP records
van Haastregt et al., 2000 [29]	Falls and mobility impairments	Not specified	Not specified	Not applicable	Not specified
Campbell et al., 1999 (Psychotropic) [30]	Falls	Not specified	Not specified	Previous hip fracture recorded at baseline only	Not specified
Campbell et al., 1999 (2-year) [15]	Falls	Not specified	Not specified	2-year follow-up study, no new incident fractures	Not specified
Campbell et al., 1997 [31]	Falls	Serious injuries, including fractures	Not specified	Serious injury component included fractures	Not specified

**Table 4:** Quality assessment and risk of bias.

Study Citation	Study Design	Assessment Tool	Random Sequence/ Selection Bias	Allocation Concealment	Blinding Participants	Blinding Assessors	Incomplete Data	Selective Reporting	Other Bias	Overall Risk
Phelan et al., 2024 [17]	RCT	Cochrane RoB 2	Low risk	Low risk	High risk	Low risk	Low risk	Low risk	Low risk	Some concerns
Marrocco et al., 2023 [18]	RCT	Cochrane RoB 2	Unclear	Unclear	High risk	Unclear	Unclear	Unclear	Unclear	High risk
Hentschke et al., 2021 [19]	RCT	Cochrane RoB 2	Unclear	Unclear	High risk	Unclear	Unclear	Unclear	Low risk	High risk
Bruce et al., 2021 [16]	RCT	Cochrane RoB 2	Low risk	Low risk	High risk	Low risk	Low risk	Low risk	Low risk	Some concerns
Bhasin et al., 2020 [20]	RCT	Cochrane RoB 2	Low risk	Low risk	High risk	Low risk	Low risk	Low risk	Low risk	Some concerns
Boongird et al., 2017 [21]	RCT	Cochrane RoB 2	Unclear	Unclear	High risk	Unclear	Unclear	Unclear	Unclear	High risk
Siegrist et al., 2016 [22]	RCT	Cochrane RoB 2	Low risk	Low risk	High risk	Unclear	Low risk	Low risk	Low risk	Some concerns
Gawler et al., 2016 [23]	RCT	Cochrane RoB 2	Low risk	Low risk	High risk	Unclear	Some concerns	Low risk	Low risk	Some concerns
Ciaschini et al., 2009 [24]	RCT	Cochrane RoB 2	Low risk	Low risk	High risk	Low risk	Low risk	Low risk	Low risk	Some concerns
Shumway-Cook et al., 2007 [25]	RCT	Cochrane RoB 2	Low risk	Unclear	High risk	Unclear	Some concerns	Some concerns	Low risk	High risk
Trivedi et al., 2003 [26]	RCT	Cochrane RoB 2	Low risk	Low risk	Low risk	Low risk	Low risk	Low risk	Low risk	Low risk
Robertson et al., 2001 (Trial II) [27]	RCT	Cochrane RoB 2	Low risk	Unclear	High risk	Some concerns	Low risk	Some concerns	Low risk	Some concerns
Robertson et al., 2001 (Trial I) [28]	RCT	Cochrane RoB 2	Low risk	Low risk	High risk	Low risk	Low risk	Low risk	Low risk	Some concerns
van Haastregt et al., 2000 [29]	RCT	Cochrane RoB 2	Low risk	Unclear	High risk	Unclear	Some concerns	Some concerns	Low risk	High risk
Campbell et al., 1999 (Psychotropic) [30]	RCT	Cochrane RoB 2	Low risk	Low risk	High risk	Some concerns	High risk	Some concerns	Low risk	High risk
Campbell et al., 1999 (2-year) [15]	Follow-up study	ROBINS-I	N/A	N/A	N/A	Some concerns	Some concerns	Low risk	Low risk	Some concerns
Campbell et al., 1997 [31]	RCT	Cochrane RoB 2	Low risk	Low risk	High risk	Low risk	Low risk	Low risk	Low risk	Some concerns

**Abbreviations**: RCT = Randomized Controlled Trial; N/A = Not Available.

**Table 5:** GRADE evidence assessment.

Outcome	Participants (Studies)	Risk of Bias	Inconsistency	Indirectness	Imprecision	Publication Bias	Certainty	Summary of Findings
Hip fractures	12,489 participants; 132 events (2 RCTs: Trivedi 2003 [26], Bruce 2021 [16])	Not serious ○	Not assessable	Not serious ○	Very serious ↓↓	Not detected ○	Very low	Limited evidence from two RCTs suggests possible hip fracture reduction (individual RR 0.80–0.87); pooled analysis not performed due to insufficient studies
Any fractures	18,519 participants; 1343 events (5 studies: Ciaschini, Siegrist, Trivedi, Bruce, and Bhasin)	Serious ↓	Very serious ↓↓	Not serious ○	Serious ↓	Not detected ○	Very low	Uncertain whether fall prevention interventions reduce any fractures (RR 0.91, 95% CI 0.72–1.14
Falls	25,000+ participants (14 RCTs)	Serious ↓	Serious ↓	Not serious ○	Not serious ○	Possibly serious ↓	Low	Fall prevention interventions may reduce falls (Large evidence base with consistent direction)
Serious Fall Injuries	8000+ participants (3 RCTs: Phelan, Bhasin, and Robertson)	Not serious ○	Serious ↓	Serious ↓	Serious ↓	Not detected ○	Low	Fall prevention interventions may reduce serious fall injuries (Heterogeneous definitions)

**Abbreviations:** RCT = Randomized Controlled Trial; RR = Risk Ratio. Symbols: ○ = no serious concern; ↓ = serious concern; ↓↓ = very serious concern.

## Data Availability

All data generated or analyzed during this study are included in this published article.

## Supplementary Materials

The following supporting information can be downloaded at: https://www.mdpi.com/article/10.3390/clinpract16030052/s1, PRISMA 2020 checklist.

## References

[1] Vaishya R., Vaish A. (2020). Falls in Older Adults are Serious. Indian J. Orthop..

[2] van der Velde N., Seppala L.J., Herrero A.C., Annweiler C., Jónsdóttir A.B., Blain H., Dionyssiotis Y., Duque S., Frith J., Francis B.N. (2025). Falls prevention in community-dwelling older adults and implementation of world falls guidelines: A call for action across Europe by the European Geriatric Medicine Society Special Interest Group on Falls and Fractures. Eur. Geriatr. Med..

[3] Peng J., Ye P., Nan B., Yan S., Li Z., Li Q., Meng R., Li Y., Hao T., Zhang L. (2025). A Fall Prevention Program Integrated in Primary Health Care for Older People in Rural China: The FAMILY Cluster Randomized Clinical Trial. JAMA.

[4] Yang Y., Komisar V., Shishov N., Lo B., Korall A.M., Feldman F., Robinovitch S.N. (2020). The Effect of Fall Biomechanics on Risk for Hip Fracture in Older Adults: A Cohort Study of Video-Captured Falls in Long-Term Care. J. Bone Miner. Res..

[5] Luo Y. (2025). Hip Fractures: Clinical, Biomaterial and Biomechanical Insights into a Common Health Challenge. Bioengineering.

[6] Sadaqa M., Németh Z., Makai A., Prémusz V., Hock M. (2023). Effectiveness of exercise interventions on fall prevention in ambulatory community-dwelling older adults: A systematic review with narrative synthesis. Front. Public Health.

[7] Meulenbroeks I., Mercado C., Gates P., Nguyen A., Seaman K., Wabe N., Silva S.M., Zheng W.Y., Debono D., Westbrook J. (2024). Effectiveness of fall prevention interventions in residential aged care and community settings: An umbrella review. BMC Geriatr..

[8] Guirguis-Blake J.M., Michael Y.L., Perdue L.A., Coppola E.L., Beil T.L. (2018). Interventions to Prevent Falls in Older Adults: Updated Evidence Report and Systematic Review for the US Preventive Services Task Force. JAMA.

[9] Montero-Odasso M., van der Velde N., Martin F.C., Petrovic M., Tan M.P., Ryg J., Aguilar-Navarro S., Alexander N.B., Becker C., Blain H. (2022). World guidelines for falls prevention and management for older adults: A global initiative. Age Ageing.

[10] Torres-Lopez R., Obradors N., Elosua R., Azagra-Ledesma R., Zwart M. (2025). Efficacy of Vitamin D Supplementation on the Risk of Falls Among Community-Dwelling Older Adults: A Systematic Review and Meta-Analysis. J. Clin. Med..

[11] Zhong Y.-J., Meng Q., Su C.-H. (2024). Mechanism-Driven Strategies for Reducing Fall Risk in the Elderly: A Multidisciplinary Review of Exercise Interventions. Healthcare.

[12] Tan L., He R., Zheng X. (2024). Effect of vitamin D, calcium, or combined supplementation on fall prevention: A systematic review and updated network meta-analysis. BMC Geriatr..

[13] Lee S., Yu S. (2020). Effectiveness of multifactorial interventions in preventing falls among older adults in the community: A systematic review and meta-analysis. Int. J. Nurs. Stud..

[14] Page M.J., McKenzie J.E., Bossuyt P.M., Boutron I., Hoffmann T.C., Mulrow C.D., Shamseer L., Tetzlaff J.M., Akl E.A., Brennan S.E. (2021). The PRISMA 2020 statement: An updated guideline for reporting systematic reviews. BMJ.

[15] Campbell A.J., Robertson M.C., Gardner M.M., Norton R.N., Buchner D.M. (1999). Falls prevention over 2 years: A randomized controlled trial in women 80 years and older. Age Ageing.

[16] Bruce J., Hossain A., Lall R., Withers E.J., Finnegan S., Underwood M., Ji C., Bojke C., Longo R., Hulme C. (2021). Fall prevention interventions in primary care to reduce fractures and falls in people aged 70 years and over: The PreFIT three-arm cluster RCT. Health Technol. Assess..

[17] Phelan E.A., Williamson B.D., Balderson B.H., Cook A.J., Piccorelli A.V., Fujii M.M., Nakata K.G., Graham V.F., Theis M.K., Turner J.P. (2024). Reducing Central Nervous System-Active Medications to Prevent Falls and Injuries Among Older Adults: A Cluster Randomized Clinical Trial. JAMA Netw. Open.

[18] Marrocco W., Galli A., Scotti S., Calabrese N., Misericordia P., Dalle Vedove A., Marrocco G., D’Ingianna A.P., Pizzini A., Fini M. (2023). A Multicomponent Primary-Care Intervention for Preventing Falls in Older Adults Living in the Community: The PREMIO Study. J. Clin. Med..

[19] Hentschke C., Halle M., Geilhof B., Landendoerfer P., Blank W., Sieber C.C., Siegrist M., Freiberger E. (2021). 24-Months Cluster-Randomized Intervention Trial of a Targeted Fall Prevention Program in a Primary Care Setting. J. Gen. Intern. Med..

[20] Bhasin S., Gill T.M., Reuben D.B., Latham N.K., Ganz D.A., Greene E.J., Dziura J., Basaria S., Gurwitz J.H., Dykes P.C. (2020). A Randomized Trial of a Multifactorial Strategy to Prevent Serious Fall Injuries. N. Engl. J. Med..

[21] Boongird C., Keesukphan P., Phiphadthakusolkul S., Rattanasiri S., Thakkinstian A. (2017). Effects of a simple home-based exercise program on fall prevention in older adults: A 12-month primary care setting, randomized controlled trial. Geriatr. Gerontol. Int..

[22] Siegrist M., Freiberger E., Geilhof B., Salb J., Hentschke C., Landendoerfer P., Linde K., Halle M., Blank W.A. (2016). Fall Prevention in a Primary Care Setting. Dtsch. Ärzteblatt Int..

[23] Gawler S., Skelton D.A., Dinan-Young S., Masud T., Morris R.W., Griffin M., Kendrick D., Iliffe S. (2016). Reducing falls among older people in general practice: The ProAct65+ exercise intervention trial. Arch. Gerontol. Geriatr..

[24] Ciaschini P.M., Straus S.E., Dolovich L.R., Goeree R.A., Leung K.M., Woods C.R., Zimmerman G.M., Majumdar S.R., Spadafora S., Fera L.A. (2009). Community-based intervention to optimise falls risk management: A randomised controlled trial. Age Ageing.

[25] Shumway-Cook A., Silver I.F., LeMier M., York S., Cummings P., Koepsell T.D. (2007). Effectiveness of a community-based multifactorial intervention on falls and fall risk factors in community-living older adults: A randomized, controlled trial. J. Gerontol. Ser. A Biol. Sci. Med. Sci..

[26] Trivedi D.P., Doll R., Khaw K.T. (2003). Effect of four monthly oral vitamin D3 (cholecalciferol) supplementation on fractures and mortality in men and women living in the community: Randomised double blind controlled trial. BMJ.

[27] Robertson M.C., Gardner M.M., Devlin N., McGee R., Campbell A.J. (2001). Effectiveness and economic evaluation of a nurse delivered home exercise programme to prevent falls. 2: Controlled trial in multiple centres. BMJ.

[28] Robertson M.C., Devlin N., Gardner M.M., Campbell A.J. (2001). Effectiveness and economic evaluation of a nurse delivered home exercise programme to prevent falls. 1: Randomised controlled trial. BMJ.

[29] van Haastregt J.C., Diederiks J.P., van Rossum E., de Witte L.P., Voorhoeve P.M., Crebolder H.F. (2000). Effects of a programme of multifactorial home visits on falls and mobility impairments in elderly people at risk: Randomised controlled trial. BMJ.

[30] Campbell A.J., Robertson M.C., Gardner M.M., Norton R.N., Buchner D.M. (1999). Psychotropic medication withdrawal and a home-based exercise program to prevent falls: A randomized, controlled trial. J. Am. Geriatr. Soc..

[31] Campbell A.J., Robertson M.C., Gardner M.M., Norton R.N., Tilyard M.W., Buchner D.M. (1997). Randomised controlled trial of a general practice programme of home based exercise to prevent falls in elderly women. BMJ.

[32] Søgaard A.J., Aga R., Holvik K., Meyer H.E. (2022). Characteristics of fallers who later sustain a hip fracture: A NOREPOS study. Osteoporos. Int..

[33] Gannon B., O’Shea E., Hudson E. (2008). Economic Consequences of Falls and Fractures among Older People. Ir. Med. J..

[34] Hagen G., Magnussen J., Tell G., Omsland T. (2020). Estimating the future burden of hip fractures in Norway. A NOREPOS study. Bone.

[35] Kjeldgaard H.K., Meyer H.E., O’Flaherty M., Apalset E.M., Dahl C., Emaus N., Fenstad A.M., Furnes O., Gjertsen J.E., Hoff M. (2022). Impact of Total Hip Replacements on the Incidence of Hip Fractures in Norway During 1999–2019. A Norwegian Epidemiologic Osteoporosis Studies (NOREPOS) Study. J. Bone Miner. Res..

[36] Dionyssiotis Y. (2012). Analyzing the problem of falls among older people. Int. J. Gen. Med..

[37] Hopewell S., Copsey B., Nicolson P., Adedire B., Boniface G., Lamb S. (2020). Multifactorial interventions for preventing falls in older people living in the community: A systematic review and meta-analysis of 41 trials and almost 20,000 participants. Br. J. Sports Med..

[38] Ojo E.O., Thiamwong L. (2022). Effects of Nurse-Led Fall Prevention Programs for Older Adults: A Systematic Review. Pac. Rim Int. J. Nurs. Res..

[39] Steinman B.A., Nguyen A.Q.D., Pynoos J., Leland N.E. (2011). Falls-Prevention Interventions for Persons Who Are Blind or Visually Impaired. Insight.

[40] Do N.M., Tolos C. (2025). Empowering Fall Prevention Through Integrated Lifestyle Medicine Strategies-From Recognition of Fall Risks to Implementation of Prevention of Falls for all in Practice. Am. J. Lifestyle Med..

[41] Yosef T., Pasco J.A., Tembo M.C., Williams L.J., Holloway-Kew K.L. (2024). Falls and fall-related injuries: Prevalence, characteristics, and treatment among participants of the Geelong Osteoporosis Study. Front. Public Health.

[42] Jennings L.A., Reuben D.B., Kim S.B., Keeler E., Roth C.P., Zingmond D.S., Wenger N.S., Ganz D.A. (2015). Targeting a high-risk group for fall prevention: Strategies for health plans. Am. J. Manag. Care.

[43] Tricco A.C., Thomas S.M., Veroniki A.A., Hamid J.S., Cogo E., Strifler L., Khan P.A., Robson R., Sibley K.M., MacDonald H. (2017). Comparisons of Interventions for Preventing Falls in Older Adults: A Systematic Review and Meta-analysis. JAMA.

[44] Langdahl B., Ferrari S., Dempster D.W. (2016). Bone modeling and remodeling: Potential as therapeutic targets for the treatment of osteoporosis. Ther. Adv. Musculoskelet. Dis..

[45] Pillay J., Gaudet L.A., Saba S., Vandermeer B., Ashiq A.R., Wingert A., Hartling L. (2024). Falls prevention interventions for community-dwelling older adults: Systematic review and meta-analysis of benefits, harms, and patient values and preferences. Syst. Rev..

[46] Wong R.M.Y., Chong K.C., Law S.W., Ho W.T., Li J., Chui C.S., Chow S.K.H., Cheung W.H. (2020). The effectiveness of exercises on fall and fracture prevention amongst community elderlies: A systematic review and meta-analysis. J. Orthop. Transl..

[47] Dautzenberg L., Beglinger S., Tsokani S., Zevgiti S., Raijmann R., Rodondi N., Scholten R., Rutjes A.W.S., Di Nisio M., Emmelot-Vonk M. (2021). Interventions for preventing falls and fall-related fractures in community-dwelling older adults: A systematic review and network meta-analysis. J. Am. Geriatr. Soc..

[48] Zhao R., Feng F., Wang X. (2017). Exercise interventions and prevention of fall-related fractures in older people: A meta-analysis of randomized controlled trials. Int. J. Epidemiol..

[49] Caristia S., Campani D., Cannici C., Frontera E., Giarda G., Pisterzi S., Terranova L., Payedimarri A.B., Faggiano F., Dal Molin A. (2021). Physical exercise and fall prevention: A systematic review and meta-analysis of experimental studies included in Cochrane reviews. Geriatr. Nurs..

