# Supplementary material for: Fall Prevention Interventions and Fracture Risk in Community-Dwelling Older Adults: A Systematic Review and Meta-Analysis
Source: Clin Pract. 2026 Feb 28;16(3):52. doi: 10.3390/clinpract16030052 (PMC13025119; doi:10.3390/clinpract16030052)
Supplement: Supplementary file 1 [file clinpract-16-00052-s001.zip › clinpract-4004248-supplementary.pdf]

PRISMA 2020 Checklist

Section and Topic	Item #	Checklist item	Location where item is reported
**TITLE**			
**Title**	1	Identify the report as a systematic review, meta-analysis, or both.	Title (p. 1): "Fall Prevention Interventions and Fracture Risk in Community-Dwelling Older Adults: A Systematic Review and Meta-Analysis"
**ABSTRACT**			
**Abstract**	2	See the PRISMA 2020 for Abstracts checklist.	Abstract (p. 1): Structured abstract with Introduction, Methods, Results, and Conclusions
**INTRODUCTION**			
**Rationale**	3	Describe the rationale for the review in the context of existing knowledge.	Section 1 (Introduction), paragraphs 1–5: Discusses fall burden, fall–fracture gap, prior evidence, and knowledge gaps
**Objectives**	4	Provide an explicit statement of the objective(s) or question(s) the review addresses.	Section 1 (Introduction), last paragraph: "The objective of this systematic review and meta-analysis is to evaluate the effectiveness of fall prevention interventions in reducing fracture outcomes…"
**METHODS**			
**Eligibility criteria**	5	Specify the inclusion and exclusion criteria for the review and how studies were grouped for the syntheses.	Section 2.2 (Eligibility Criteria): Inclusion criteria (population, intervention, outcomes) and exclusion criteria detailed
**Information sources**	6	Specify all databases, registers, websites, organisations, reference lists and other sources searched or consulted to identify studies. Specify the date when each source was last searched or consulted.	Section 2.1 (Study Design and Search Strategy): MEDLINE (PubMed), Scopus, Cochrane CENTRAL, Web of Science, Google Scholar; searched from inception through 7 June 2025
**Search strategy**	7	Present the full search strategies for all databases, registers and websites, including any filters and limits used.	Section 2.1: Full Boolean search strategy with key terms and concepts presented in text

Section and Topic	Item #	Checklist item	Location where item is reported
**Selection process**	8	Specify the methods used to decide whether a study met the inclusion criteria of the review, including how many reviewers screened each record and each report retrieved, whether they worked independently, and if applicable, details of automation tools used in the process.	Section 2.3 (Study Selection and Data Extraction): Two reviewers (Y.J.A and M.A.A) independently screened; disagreements resolved by third reviewer (A.Y.A.); Cohen's kappa reported
**Data collection process**	9	Specify the methods used to collect data from reports, including how many reviewers collected data from each report, whether they worked independently, any processes for obtaining or confirming data from study investigators, and if applicable, details of automation tools used in the process.	Section 2.3: Four reviewers (Y.J.A, L.A.Alzahrani, N.I.A, S.M.A) working in pairs using standardized extraction form; disagreements resolved by consensus (A.Y.A.)
**Data items**	10a	List and define all outcomes for which data were sought. Specify whether all results that were compatible with each outcome domain in each study were sought, and if not, the methods used to decide which results to collect.	Section 2.3 and Tables 1–3: Extracted variables listed (study design, sample size, age, sex, intervention type, duration, fracture outcomes, ascertainment methods)
	10b	List and define all other variables for which data were sought. Describe any assumptions made about any missing or unclear information.	Section 2.3: Standardized extraction form covering study characteristics, population demographics, intervention details, and outcome measurements
**Study risk of bias assessment**	11	Specify the methods used to assess risk of bias in the included studies, including details of the tool(s) used, how many reviewers assessed each study and whether they worked independently, and if applicable, details of automation tools used in the process.	Section 2.4 (Quality Assessment and Risk of Bias): Cochrane RoB 2 for RCTs (5 domains); ROBINS-I for non-randomized studies; two independent assessors; inter-rater agreement calculated
**Effect measures**	12	Specify for each outcome the effect measure(s) used in the synthesis or presentation of results.	Section 2.5 (Statistical Analysis): Risk ratios (RRs) with 95% CIs; random-effects DerSimonian–Laird method; NNT, ARR, and RRR calculated where appropriate
**Synthesis methods**	13a	Describe the processes used to decide which studies were eligible for each synthesis.	Sections 3.5–3.6: Studies grouped by fracture type (hip fractures: 2 RCTs; any fractures: 5

Section and Topic	Item #	Checklist item	Location where item is reported
			studies); exclusion of composite endpoints without extractable fracture data explained
	13b	Describe any methods required to prepare the data for presentation or synthesis, such as handling of multi-arm trials.	Section 2.5 and Table 1 footnotes: Gawler 2016 three-arm trial handling described; Campbell 1999 multiple-arm trial components separated
	13c	Describe any methods used to tabulate or visually display results of individual studies and syntheses.	Forest plots (Figure 2), funnel plots (Figure 4), leave-one-out influence plots (Figure 3), and summary Tables 1–5
	13d	Describe any methods used to synthesize results and provide a rationale for the choice(s). If meta-analysis was performed, describe the model(s), method(s) to identify the presence and extent of statistical heterogeneity, and software package(s) used.	Section 2.5: Random-effects DerSimonian–Laird; heterogeneity assessed via Cochran's Q, I^2, τ^2; HKSJ adjustment; RStudio with meta and metafor packages
	13e	Describe any methods used to explore possible causes of heterogeneity among study results.	Section 2.5: Subgroup analyses a priori by intervention type, study design, participant characteristics, risk of bias; sensitivity analyses including leave-one-out and HKSJ adjustment
	13f	Describe any sensitivity analyses conducted to assess robustness of the synthesized results.	Section 3.8 (Subgroup and Sensitivity Analysis): HKSJ adjustment, leave-one-out analysis (Figure 3), influence diagnostics
**Reporting bias assessment**	14	Describe any methods used to assess risk of reporting biases across studies.	Section 2.5 and 3.9: Funnel plot visual inspection (Figure 4); formal statistical testing limited by small number of studies (n = 5)
**Certainty assessment**	15	Describe any methods used to assess certainty (or confidence) in the body of evidence for an outcome.	Section 2.6 (Certainty of Evidence Assessment): GRADE approach; rated high/moderate/low/very low across 5 domains (risk of bias, inconsistency, indirectness, imprecision, publication bias)

Section and Topic	Item #	Checklist item	Location where item is reported
**RESULTS**			
**Study selection**	16a	Describe the results of the search and selection process, from the number of records identified in the search to the number of studies included in the review, ideally using a flow diagram.	Section 3.1 and Figure 1 (PRISMA flowchart): 429 records identified → 26 duplicates removed → 248 removed before screening → 155 screened → 138 excluded → 17 studies included
	16b	Cite studies that might appear to meet the inclusion criteria, but which were excluded, and explain why they were excluded.	Section 3.1: Exclusion reasons detailed (155 records not retrieved with reasons; 138 reports excluded after full-text review)
**Study characteristics**	17	Cite each included study and present its characteristics.	Table 1 (Study characteristics and participant demographics): All 17 studies cited with design, sample size, age, sex, country, setting, intervention, duration, primary endpoint
**Risk of bias in studies**	18	Present assessments of risk of bias for each included study.	Table 4 (Quality assessment and risk of bias): RoB 2 domain-level assessments for all 17 studies across 5 domains with overall judgment
**Results of individual studies**	19	For all outcomes, present, for each study: (a) summary statistics for each group and (b) an effect estimate and its precision.	Tables 1 and 3; Sections 3.5–3.6: Individual RRs with 95% CIs reported for each study (e.g., Trivedi RR 0.87 [0.49–1.55]; Bruce RR 0.80 [0.62–1.04])
**Results of syntheses**	20a	For each synthesis, briefly summarise the characteristics and risk of bias among contributing studies.	Sections 3.5–3.6: Study characteristics and risk of bias summarized for each analysis group (hip fractures: 2 RCTs; any fractures: 5 studies)
	20b	Present results of all statistical syntheses conducted. If meta-analysis was done, present for each the summary estimate and its precision and measures of statistical heterogeneity. If other methods of synthesis were used, present the results as structured in the planned methods.	Section 3.6 and Figure 2: Pooled RR 0.91 (95% CI 0.72–1.14); $I^2 = 65\%$; $\tau^2 = 0.03$; Q = 11.40, df = 4, p = 0.02. Hip fractures: qualitative synthesis (insufficient for pooling)

Section and Topic	Item #	Checklist item	Location where item is reported
	20c	Present results of all investigations of possible causes of heterogeneity among study results.	Section 3.8: Leave-one-out analysis showing Bruce et al. as main heterogeneity driver (I^2 drops to 0% when excluded); HKSJ adjustment widened CIs (0.63–1.31)
	20d	Present results of all sensitivity analyses conducted to assess the robustness of the synthesized results.	Section 3.8 and Figure 3: HKSJ adjustment (RR 0.91, CI 0.63–1.31); leave-one-out influence analysis; results remained non-significant across all sensitivity analyses
**Reporting biases**	21	Present assessments of risk of reporting biases across studies for each synthesis assessed.	Section 3.9 and Figure 4: Funnel plot for any fractures (5 studies); visual inspection showed reasonable distribution; formal testing limited by small study count
**Certainty of evidence**	22	Present assessments of certainty (or confidence) in the body of evidence for each outcome assessed.	Section 3.7 and Table 5 (GRADE evidence assessment): Hip fractures – very low; Any fractures – very low; Falls – low; Serious fall injuries – very low
**DISCUSSION**			
**Discussion**	23a	Provide a general interpretation of the results in the context of other evidence.	Section 4 (Discussion), paragraphs 1–4: Results contextualized against prior literature [8,15,43,45–49]; fall–fracture disconnect discussed
	23b	Discuss any limitations of the evidence included in the review.	Section 4, paragraphs 10–14: Five limitations detailed (small study count, intervention heterogeneity, methodological concerns, outcome measurement variability, publication bias)
	23c	Discuss any limitations of the review processes used.	Section 4, paragraphs 10–14: Language restrictions, search strategy limitations,

Section and Topic	Item #	Checklist item	Location where item is reported
			inability to perform formal publication bias testing acknowledged
	23d	Discuss implications of the results for practice, policy, and future research.	Section 4, paragraphs 15–19 and Section 5 (Conclusions): Integrated fall + bone health strategies recommended; standardized outcome definitions urged; future RCTs with fracture primary endpoints called for
**OTHER INFORMATION**			
**Registration and protocol**	24a	Provide registration information for the review, including register name and registration number, or state that the review was not registered.	Section 2.1: "This systematic review was not prospectively registered in PROSPERO"
	24b	Indicate where the review protocol can be accessed, or state that a protocol was not prepared.	Section 2.1: No separate protocol document referenced; methods described in full
	24c	Describe and explain any amendments to information provided at registration or in the protocol.	Not applicable (review was not registered)
**Support**	25	Describe sources of financial or non-financial support for the review, and the role of the funders or sponsors in the review.	Funding statement: "This research received no external funding"
**Competing interests**	26	Declare any competing interests of review authors.	Conflicts of Interest: "The authors declare no conflicts of interest"
**Availability of data, code, and other materials**	27	Report which of the following are publicly available and where they can be found: template data collection forms; data extracted from included studies; data used for all analyses; analytic code; any other materials used in the review.	Data Availability Statement: "All data generated or analyzed during this study are included in this published article"

From: *Page MJ, McKenzie JE, Bossuyt PM, et al. The PRISMA 2020 statement: an updated guideline for reporting systematic reviews.* BMJ 2021;372:n71. doi: 10.1136/bmj.n71.

For more information, visit: http://www.prisma-statement.org/
